# Anomalous origin of the fetal pulmonary artery

**DOI:** 10.3389/fped.2023.1204070

**Published:** 2023-06-29

**Authors:** Qiong Huang, Wen Ling, Qiumei Wu, Shan Guo, Tingting Dang, Hong Ma, Biying Huang, Chunxia Chen, Min Liu, Xiuqing Qiu, Zongjie Weng

**Affiliations:** ^1^ Department of Medical Ultrasonics, Fujian Maternity and Child Health Hospital, College of Clinical Medicine for Obstetrics & Gynecology and Pediatrics, Fujian Medical University, Fuzhou, China; ^2^ Department of Pathology, Fujian Maternity and Child Health Hospital, College of Clinical Medicine for Obstetrics & Gynecology and Pediatrics, Fujian Medical University, Fuzhou, China; ^3^ Department of Imaging, Fujian Maternity and Child Health Hospital, College of Clinical Medicine for Obstetrics & Gynecology and Pediatrics, Fujian Medical University, Fuzhou, China; ^4^ Department of Obstetrics & Gynecology, Fujian Maternity and Child Health Hospital, College of Clinical Medicine for Obstetrics & Gynecology and Pediatrics, Fujian Medical University, Fuzhou, China

**Keywords:** fetus, prenatal ultrasound, pulmonary artery sling, anomalous origin of the unilateral pulmonary artery, unilateral absence of the pulmonary artery

## Abstract

**Objectives:**

This study aims to investigate the efficacy of prenatal ultrasonography in diagnosing the anomalous origin of the fetal pulmonary artery (AOFPA).

**Methods:**

A total of 26 AOFPA cases were retrospectively analyzed from January 2014 to January 2023. The features of the AOFPA were characterized by comparing the prenatal ultrasonic data with the results of anatomical casting after pregnancy termination or postnatal imaging and surgical intervention. Missed diagnoses and misdiagnoses were expounded.

**Results:**

Of the 26 AOFPA cases, there were 13 cases of pulmonary artery sling, 8 cases of anomalous origin of the unilateral pulmonary artery, and five cases of unilateral absence of the pulmonary artery; 17 cases received pathological anatomy and casting after pregnancy termination, and nine cases were confirmed by postnatal imaging and surgery. Nineteen cases were accurately prenatally diagnosed (19/26, 73.1%), and seven cases were missed or misdiagnosed (7/26, 26.9%).

**Conclusions:**

Prenatal ultrasonography has a favorable diagnostic efficacy for anomalous origin of the fetal pulmonary artery. The absence of either the left or right pulmonary artery from the image of pulmonary artery bifurcation may indicate origin abnormalities of the pulmonary artery in fetuses, which signifies the necessity to detect the abnormal origin of the pulmonary artery on the affected side and other potential intracardiac malformation complications.

## Introduction

The anomalous origin of the pulmonary artery (AOPA) is a rare congenital cardiovascular malformation, including pulmonary artery sling (PAS), anomalous origin of the unilateral pulmonary artery (AOUPA), and unilateral absence of the pulmonary artery (UAPA) (1). The condition may occur independently or be complicated with other cardiovascular malformations. Due to the disparities in pathologic anatomy, its prognosis varies greatly for different patients (2, 3). As the right pulmonary artery sling is rare and may occur only in the presence of visceral isomerism (4), PAS usually refers to the left pulmonary artery sling, in which the left pulmonary artery originates from the posterior wall of the right pulmonary artery and travels between the esophagus and trachea to form a vascular ring to reach the left hilus (5). PAS is usually associated with tracheobronchial abnormalities and other congenital heart defects (6). In AOUPA, the anomalous pulmonary artery branch originates from the ascending aorta, with an incidence of about 0.12% (7), and the other pulmonary artery branch continues with the main pulmonary artery. Anatomically, AOUPA can be divided into the anomalous origin of the right pulmonary artery branch from the aorta (AORPA) and the anomalous origin of the left pulmonary artery branch from the aorta (AOLPA) (1). It can be further divided into proximal and distal types according to the distance between the location of anomalous pulmonary artery origin and the aortic valve: (1) the proximal type accounts for 85% of AOUPA, with the opening of the pulmonary artery located close to the aortic valve; and (2) the distal type accounts for 15% of AOUPA, with the opening of the pulmonary artery located near the beginning of the innominate artery (1, 8). Studies have documented different embryonic developmental mechanisms for AOLPA and AORPA and for the proximal and distal types of AOUPA (9, 10). A recent study speculates that the distal type of AOUPA might be a manifestation of UAPA during the fetal period (3). The latter is a rare form of congenital pulmonary vascular dysplasia, with an incidence of about 1 in 200,000 (11), referring to unilateral proximal pulmonary artery occlusion or unilateral absence of the intrapericardial segment of the pulmonary artery (12).

To date, an accurate diagnosis of abnormalities in fetal pulmonary artery origin remains challenging. Due to its rare occurrence, AOPA runs an extremely high risk of missed diagnosis or misdiagnosis (3, 13). Other studies have demonstrated that infants with AOPA report a grave mortality rate if postnatal surgery is not performed in due time (13–15). Some selected cases are controversial in the partial literature. Therefore, an accurate prenatal diagnosis of AOPA is essential for antenatal counseling, perinatal management, and postnatal care. This study aims to improve the accuracy of prenatal diagnosis of AOPA by retrospectively analyzing the prenatal ultrasound images, postnatal anatomical manifestations, and postnatal examinations of AOPA cases.

## Materials and methods

### Study population

From January 2014 to January 2023, 103,227 fetuses underwent systematic ultrasonography and 68,198 fetuses were examined at 11–13^+6^ weeks. A total of 3,867 cases were identified for congenital heart disease, and 26 AOPA cases were confirmed by pathological autopsy, postnatal imaging, or surgery, with the pregnancy age ranging from 23 to 40 years and the gestation age ranging from 12.7 to 35.6 weeks. The study was approved by the Ethics Committee of Fujian Maternity and Child Health Hospital, Fujian Medical University (2014FY110700), and informed consent was obtained from the families. In pregnancy termination cases, the families signed an informed consent for autopsy or casting verification.

### Fetal echocardiography

Philips IU22 and GE Voluson S8, E8, and E10 ultrasound diagnostic instruments were applied, with the probe frequency set at 4.0–8.0 MHz. According to the International Society of Ultrasound in Obstetrics and Gynecology (ISUOG) fetal cardiac screening guidelines (16), the fetal heart was scanned by segmental sequence analysis. For suspected AOPA cases, the three-vessel series section (three-vessel section, trivascular-tracheal section, trivascular-pulmonary artery bifurcation section) should be used for observation, and the left/right ventricular outflow tract, ascending aorta, pulmonary artery trunk and its branches, ductus arteriosus (DA), etc. should be explored. In the case of a suspected PAS, the coronal section of the trachea was scanned to reveal the course and inner diameter of the trachea and the details of tracheal stenosis.

### Neonatal echocardiography

Postnatal echocardiography was performed with a Philips EPIQ 7C and IE Elite ultrasound diagnostic instrument, with the probe frequency set at 3.0–8.0 MHz. According to the American Society of Echocardiography (ASE) pediatric echocardiography guidelines (17), the heart was comprehensively scanned by segmental analysis to observe the origin, internal diameter, and blood flow of the pulmonary artery and its branches. The development of other cardiovascular structures was also evaluated.

### Computed tomography angiography

Computed tomography angiography (CTA) was performed with a GE revolution CT (256 slices) for infants with suspected AOPA. Iopromide was injected according to the infant’s body weight (1.5 mL/kg). For infants with poor cooperation during the examination, chloral hydrate was applied. The orthostatic image was scanned in the supine position, and the region of interest was placed in the pulmonary trunk. After 5–6 s of injection, the exposure scan began when the CT value reached the threshold (70 Hu), with the following parameters: thickness 0.625 mm, exposure time 0.6–1 s, and voltage 80 kV. The observed data were postprocessed with the Advantage Windows 4.6 workstations, including volume rendering (VR) reconstruction, maximum intensity project (MIP) reconstruction, and three-dimensional (3D) tracheobronchial reconstruction, to reveal the shape of the bronchi and the movement of blood vessels more intuitively and three-dimensionally.

### Dissection and casting

Abnormal specimens of fetal pulmonary artery origin were mainly dissected by combining *in situ* observation and *ex vivo* immobilization. The specific steps are as follows: (1) after the removal of the thymus, the heart and large blood vessels were exposed and the pericardium was removed; (2) *in situ* observation of the heart was proceeded, focusing on (a) the position, axis, size, and shape of the heart; (b) the external shape of the atria and ventricles; (c) the location and size of the aorta; and (d) the size and continuity of the aortic arch, ductus arteriosus, and descending aorta after the displacement of the lungs; (3) the heart–lung tissue was removed and fixed with formalin liquid; (4) the fixed heart specimen was autopsied along the direction of blood flow to reveal the atrium, ventricle, atrioventricular septum, valve and the opening position, inner diameter, branched blood vessel, and direction of the aorta. For smaller hearts, abnormalities were observed by removing part of the atrioventricular wall or blood vessel wall. Cardiovascular casting was performed by the combination of the thoracic and abdominal cavity casting, observing the following three steps: (1) the blood vessels were rinsed with normal saline until the color of the heart and lungs turned lighter; (2) the casting agent was prepared; (3) after perfusion with the casting agent, the organs in the chest and abdomen were removed when the specimen hardened and placed in the potassium hydroxide solution for corrosion, and the molding was completed.

### Statistical analysis

The data were analyzed with SPSS 25.0. The prenatal ultrasound results were compared with the findings of postnatal imaging, surgical results, pathological anatomy, or casting. The measurement data were expressed as *x* ± *s*, and the counting data were expressed as frequency or percentage.

## Results

The 26 cases of abnormal fetal pulmonary artery origin, including 13 PAS cases, eight AOUPA cases, and five UAPA cases, are summarized in Table 1. Of them, 17 cases were confirmed by pathological autopsy after pregnancy termination and nine cases were confirmed by postnatal echocardiography, surgery, or CTA. Nineteen cases (19/26, 73.1%) were accurately diagnosed by prenatal ultrasonography, and seven cases (7/26, 26.9%) were missed or misdiagnosed.

**Table 1:** Prognosis of 26 cases of fetal pulmonary artery origin combined with intracardiac malformations.

	Number	Associated with major intracardiac malformations	Outcome	
			Termination	Parturition
PAS	13	TOF 1, right pulmonary artery dysplasia 1, HLHS 1	6	7
AOPA	8	TOF 1, Berry 1	6	2
UAPA	5	TOF 3, APVS1	5	0
Total	26		17	9

PAS, pulmonary artery sling; UAPA, unilateral absence of the pulmonary artery; AOPA, anomalous origin of unilateral pulmonary artery; TOF, tetralogy of Fallot; APVS, absent pulmonary valve syndrome; PS, pulmonary artery stenosis; HLHS, hypoplastic left heart syndrome.

### Prenatal findings of the anomalous origin of the pulmonary artery

The prenatal ultrasonography indicated that the absence of the typical bifurcation structure in the section of pulmonary artery bifurcation was a common ultrasound manifestation of AOPA. The main pulmonary artery continued to be only one pulmonary artery. Details of the PAS cases are shown in Table 2. The ultrasound images of PAS showed that the right pulmonary artery extended directly from the main pulmonary artery and the left pulmonary artery originated from the right pulmonary artery and formed a “C”-shaped vascular ring from right to left behind the trachea and traveled between the trachea and esophagus to the left hilus. Among the 13 PAS cases, five cases (5/13, 38.5%) had different degrees of compression and narrowing of the trachea, in which one reported an enlarged right lung, with an enhanced echo and left deviation of the heart axis, due to severe stenosis of the right bronchi; three cases underwent fetal cardiac ultrasonography during the early pregnancy (11 w–13^+6^ w), in which one case (Case 4) showed no apparent abnormalities; one case (Case 3) reported a right shift in the heart position, with an enlarged volume of the left lung and a slightly enhanced echo; and one case (Case 5) showed that the left pulmonary artery originated from the right pulmonary artery and bypassed behind the trachea at 12^+5^ weeks, diagnosed as PAS. Case 3 was confirmed as PAS during the middle and late pregnancy. In the same PAS population, there were five isolated PAS cases (5/13, 38.5%) and eight cases (8/13, 61.5%) complicated with other intracardiac malformations, including ventricular septal defect (VSD), permanent left superior vena cava (PLSVC), pulmonary artery dysplasia, coarctation of the aorta (CoA) and tetralogy of Fallot (TOF).

**Table 2:** Associated malformation and prognosis of PAS.

Case	Gestational age (weeks)	Associated with intracardiac malformations	Trachea (inner diameter)	Outcome	Characteristics of the case
1	23^+5^	VSD	Pressed (0.26 cm)	Termination, dissection	Severely narrow right bronchi caused an enlarged right lung and enhanced echo
2	30^+6^	VSD	Pressed (0.08 cm)	Cervical opening, urgent labor, fetal death in utero	/
3	17^+2^	Right pulmonary artery dysplasia	/	Full-term delivery, Apgar 10′, confirmed by postnatal review and surgery	Reflected the integrated prenatal and postnatal management of PAS
4	25^+3^	PLSVC	Pressed (0.08 cm)	Termination, dissection	Examination at 11–13^+6^ weeks showed no significant abnormalities
5	12^+5^	HLHS MA AA, PLSVC	/	Termination, dissection	Detected in early pregnancy, with NT thickening (2.8 mm), the blood flow spectrum of DV exhibited a reverse a-wave
6	29^+4^	/	Slightly compressed	Termination, dissection	/
7	29^+1^	Mild regurgitation of the TV	Pressed (0.13 cm)	Full-term delivery, Apgar 10′	/
8	26^+2^	Slightly narrower aorta	/	Termination, vascular casting	/
9	24	/	/	Parturition, confirmed by postnatal echocardiography and surgery	/
10	27^+5^	TOF	/	Parturition, confirmed by postnatal echocardiography	One of the twins of the double chorionic and double amniotic sac was abnormal
11	23^+2^	PLSVC	/	Parturition, Apgar 9′, confirmed by postnatal echocardiography	One of the twins of the double chorionic and double amniotic sac, missed case
12	23	/	/	Parturition, Apgar 10′, confirmed by postnatal echocardiography	One of the twins of the double chorionic and double amniotic sac, missed case
13	22^+5^	/	/	Full-term delivery, Apgar 10′, confirmed by postnatal echocardiography	Missed case

PAS, pulmonary artery sling; TOF, tetralogy of Fallot; PLSVC, persistent left superior vena cava; HLHS, hypoplastic left heart syndrome; MA, mitral atresia; AA, aortic atresia; NT, nuchal translucency; PDA, patent ductus arteriosus; ASD, atrial septal defect; DV, ductus venosus; TV, tricuspid valve.

Of the eight AOUPA cases, ultrasonography revealed that the main pulmonary artery only branched out the left (or right) pulmonary artery, with the right (or left) pulmonary artery originating from the proximal ascending aorta (closer to the aortic valve) or distal ascending aorta (near the beginning of the innominate artery), in which seven cases reported an abnormality in the origin of the right pulmonary artery and one case displayed an abnormal origin of the left pulmonary artery (Table 3). Of the seven AORPA cases, stenosis of the right pulmonary artery was found in two cases, with the right pulmonary arteries originating near the innominate artery; except for three isolated cases, complications with other intracardiac structural abnormalities (such as VSD, CoA, interruption of the aortic arch, mitral valve atresia, etc.) were shown in four cases, in which one case was coupled with aortic-pulmonary window, interruption of the aortic arch, intact ventricular septum and diagnosed as Berry syndrome. The remaining AOLPA case was complicated by TOF and the right aortic arch with mirror branches.

**Table 3:** Associated malformation and prognosis of AOPA.

Case	Gestational age (weeks)	Affected PA	Associated with intracardiac malformations	Outcome	Characteristics of the case
1	25^+4^	R	/	Termination, dissection	/
2	23^+6^	R	/	Termination, dissection	/
3	24^+6^	L	TOF, right aortic arch with mirrored branches, left innominate vein under the aortic arch	Termination, dissection	The left pulmonary artery emanated from the left side of the aortic root and crossed with the right pulmonary artery to the left lung
4	35^+4^	R	VSD	Termination, dissection	/
5	24^+2^	R	Dexiocardia, CoA, IAA, VSD, MA, tricuspid valve dysplasia with severe regurgitation	Termination, dissection	Associated with multiple intracardiac malformations and missed diagnosis in the outer hospital
6	23^+5^	R	/	Full-term delivery, Apgar 10′. The child is currently 5 years old and has not undergone any treatment	The initial part of the right pulmonary artery originating from the ascending aorta was narrowed and progressively developed into atresia
7	23	R	APW, IAA	Termination, dissection	/
8	24^+3^	R	Slightly thinner aortic isthmus	Cesarean section at 32 weeks, Apgar 8′. The child is currently 1^+^ month old and is still being followed	One of the twins of the monochorionic double amniotic sac. The initial part of the right pulmonary artery was narrowed and progressively developed into atresia

AOPA, anomalous origin of unilateral pulmonary artery; LINV, left innominate vein; VSD, ventricular septal defect; CoA, coarctation of the aorta; IAA, interruption of the aortic arch; MA, mitral atresia; APW aortopulmonary window.

All five UAPA cases reported an absence of the left pulmonary artery (Table 4), only with the right pulmonary artery directly extending from the main pulmonary artery in the section of the pulmonary artery bifurcation. The distal pulmonary artery and intrapulmonary blood flow at the left hilus were tracked, revealing that the left pulmonary artery was connected to the innominate artery or the transverse arch of the aorta via the ductus arteriosus. Of the five UAPA cases, three were concomitant with TOF, in which one case reported both TOF and absent pulmonary valve syndrome (APVS).

**Table 4:** Associated malformation and prognosis of UAPA.

Case	Gestational age (weeks)	Affected PA	Associated with intracardiac malformations	Outcome	The connection of the pulmonary artery on the affected side
1	26^+4^	L	TOF	Termination, dissection	Connected to the lower right side of the transverse arch of the aorta by DA
2	22	L	Right aortic arch with mirrored branches	Termination, dissection	Connected to the LINA by DA; misdiagnosed as AOPA
3	22^+5^	L	TOF, APVS	Termination, vascular casting	Connected to the transverse arch of the aorta by DA, misdiagnosed as AOPA
4	26	L	TOF	Termination, dissection	Connected to the transverse arch of the aorta by DA, misdiagnosed as AOPA
5	24^+4^	L	Right aortic arch with mirrored branches	Termination, dissection	Connected to the LINA by DA

UAPA, unilateral absence of the pulmonary artery; AOPA, anomalous origin of unilateral pulmonary artery; TOF, tetralogy of Fallot; APVS, absent pulmonary valve syndrome; LINA, left innominate artery; DA, ductus arteriosus.

### Prognosis of the anomalous origin of the pulmonary artery

**Figure 1:**
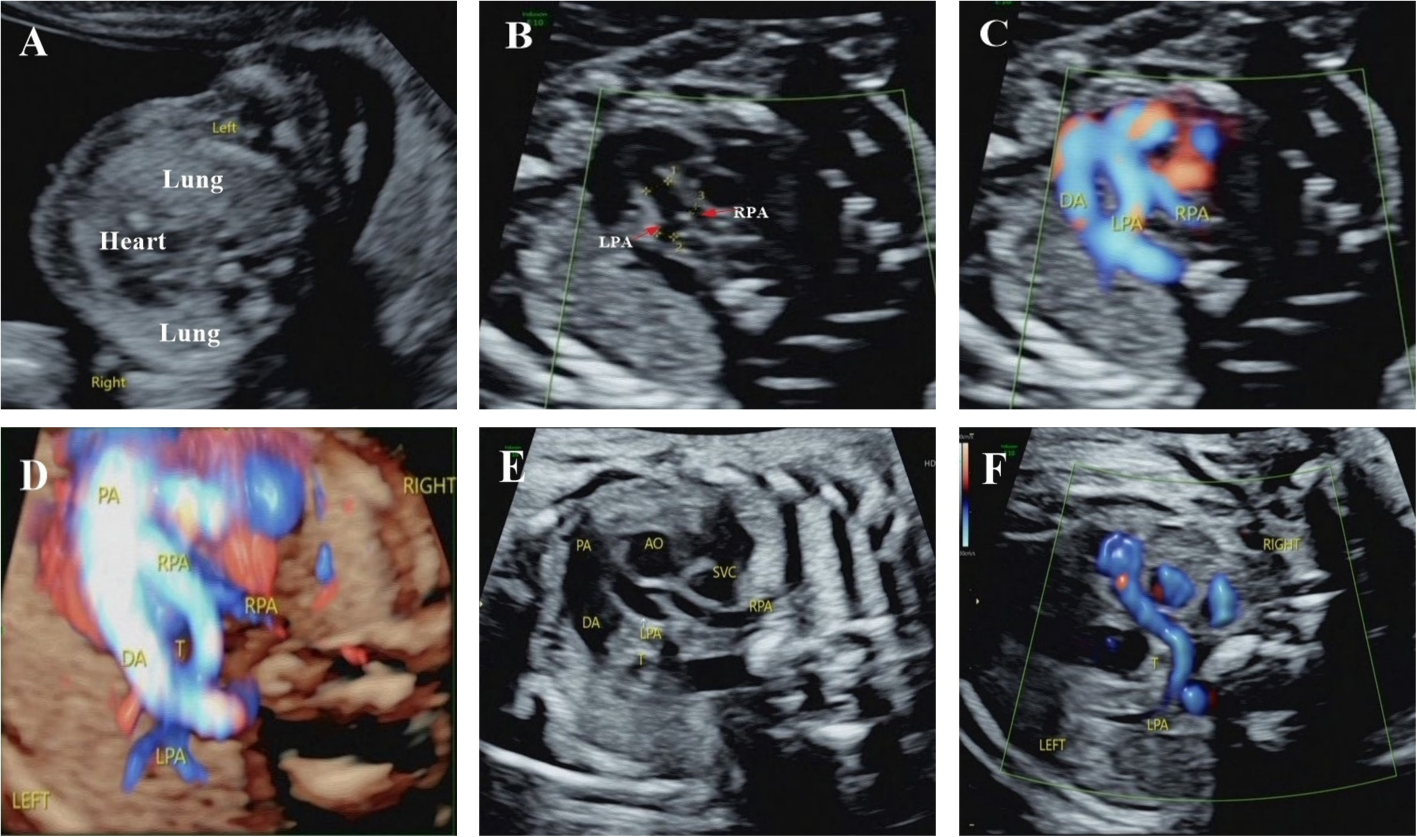
Integrated prenatal and postnatal management of PAS (prenatal portion). (**A**) Ultrasound examination at 13^+4^ weeks showed that the heart shifted to the right and the volume of the left lung increased compared with the right lung. (**B**–**D**) Ultrasound detected PAS at 17^+2^ weeks. Ultrasound examination showed that the left pulmonary artery originated from the right pulmonary artery, bypassed behind the trachea from right to left, and the right pulmonary artery had a narrow inner diameter. These suggested PAS and dysplastic right pulmonary artery. (**E,F**) Ultrasound examination at 30 weeks confirmed the same diagnosis. PA, pulmonary atresia; DA, ductus arteriosus; AO, aorta; LPA, left pulmonary artery; RPA, right pulmonary artery; SVC, superior vena cava; T, trachea.

**Figure 2:**
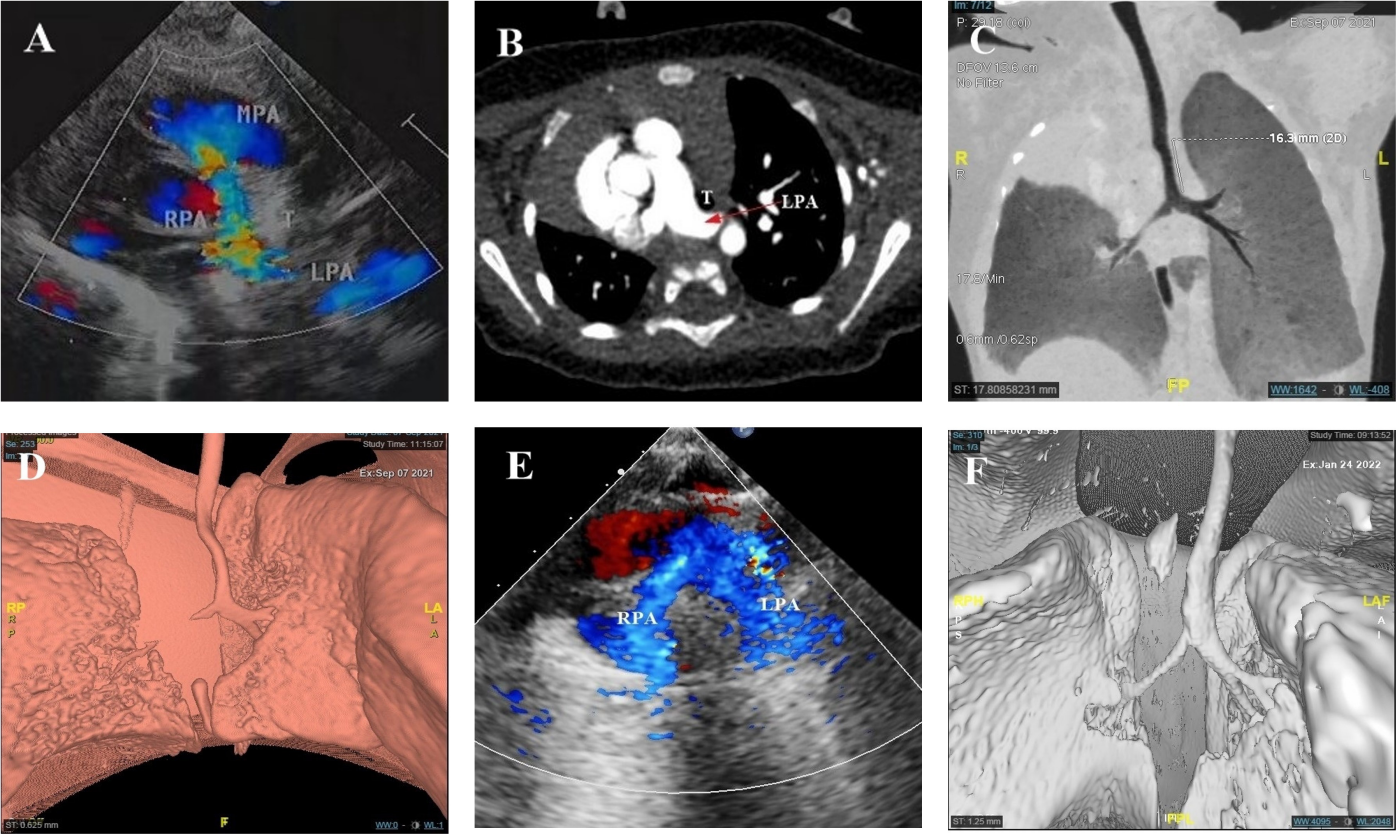
Integrated prenatal and postnatal management of PAS (postpartum portion). (**A,B**) Neonatal echocardiography and CTA showed PAS. (**B**–**D**) Minimum density projection and 3D tracheobronchial reconstruction reported long-term stenosis in the middle and upper long segment of the airway, the stenotic lower trachea, and the stenotic trachea above the carina. (**E**) Postoperative echocardiography showed that the left and right pulmonary arteries originated from the main pulmonary artery. (**F**) 3D tracheobronchial reconstruction indicated a significant improvement in tracheal stenosis when compared with the preoperative period.

Among the 13 PAS cases, five patients were re-examined for PAS by postnatal echocardiography and received no intervention due to a lack of clinical symptoms and two cases were confirmed as PAS by surgery, in which one underwent an integrated prenatal (Figure 1) and postnatal (Figure 2) management—a dynamic tracking and observation covering the early pregnancy, middle, and late pregnancy, postnatal (preoperative) period, surgery, and postoperative care. In this particular patient, signs of PAS were suspected during early pregnancy and confirmed during mid-to-late pregnancy review and postnatal echocardiography/CTA review. The patient received tracheoplasty and pulmonary artery sling surgery and recovered favorably. Of the 13 PAS cases, six cases received pregnancy termination, and the pathological anatomy and casting evidenced that the left pulmonary artery originated from the right pulmonary artery, passed by the right side of the trachea, and extended between the trachea and the esophagus into the left hilus.

**Figure 3:**
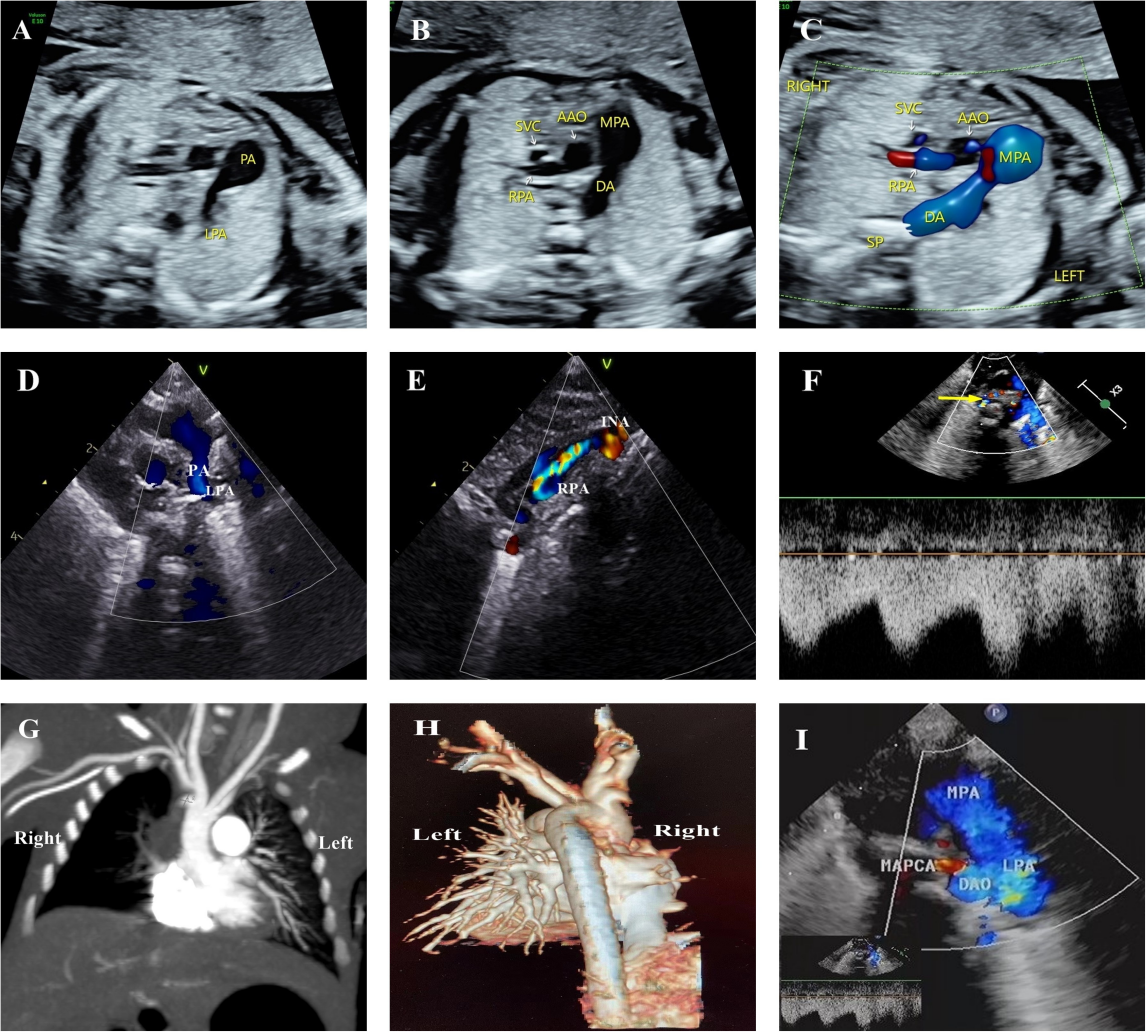
Progressive atresia of the right pulmonary artery originating from the ascending aorta in one of twins. (**A**–**C**) Main pulmonary artery continued only as the left pulmonary artery and ductus arteriosus. The right pulmonary artery originated from the ascending aorta, and a crest-like protrusion was seen at the opening of the right pulmonary artery at 24^+3^ weeks, reporting consistent results at 27^+5^ weeks and 31^+3^ weeks. (**D**, **E**) Echocardiographic review on the 1st postnatal day was consistent with prenatal results, with the right pulmonary artery originating from the lateral wall of the ascending aorta in the vicinity of the opening of the innominate artery. The course was tortuous, with the color Doppler image indicating colorful blood flow signals. (**F**) Cardiac ultrasound on the 25th postnatal day revealed a thickening of the main pulmonary artery and left pulmonary artery, and the small collateral vessels originating from the descending aorta (indicated with a yellow arrow). (**G,H**) Postnatal CTA examination and VR reconstruction only showed the left pulmonary artery branching from the main pulmonary artery, with the right pulmonary artery absent. However, no collateral vessels from the descending aorta were seen. (**I**) Repeated echocardiography on the 30th postnatal day did not expose the right pulmonary artery but revealed thicker collateral blood vessels from the aortic arch and descending aorta. MAPCA, major aortopulmonary collaterals.

Of the eight AOUPA cases, two AORPA cases reported stenosis at the beginning of the right pulmonary artery and were presented with the right pulmonary atresia after birth, with one case (Case 6 in Table 3) showing a blood supply for the right lung via collateral vessels from the descending aorta by postnatal ultrasonography and CTA. For the other case (Case 8 in Table 3), a narrowed right pulmonary artery originating from the ascending aorta (near the innominate artery) was found on the first postnatal day by ultrasonography and pulmonary atresia was evident on the 25th postnatal day, accompanied by small collateral blood vessels from the descending aorta. On the 26th postnatal day, CTA was performed, which revealed no right pulmonary artery and no collateral blood vessels in VR reconstruction. However, on the 30th postnatal day, an ultrasound examination reported thicker collateral blood vessels (Figure 3). To date, these two cases have no clinical symptoms and are still followed. The remaining six cases received pregnancy termination and the pathological anatomy precisely located the origin of the branch of the pulmonary artery at the root of the ascending aorta or near the beginning of the innominate artery.

**Figure 4:**
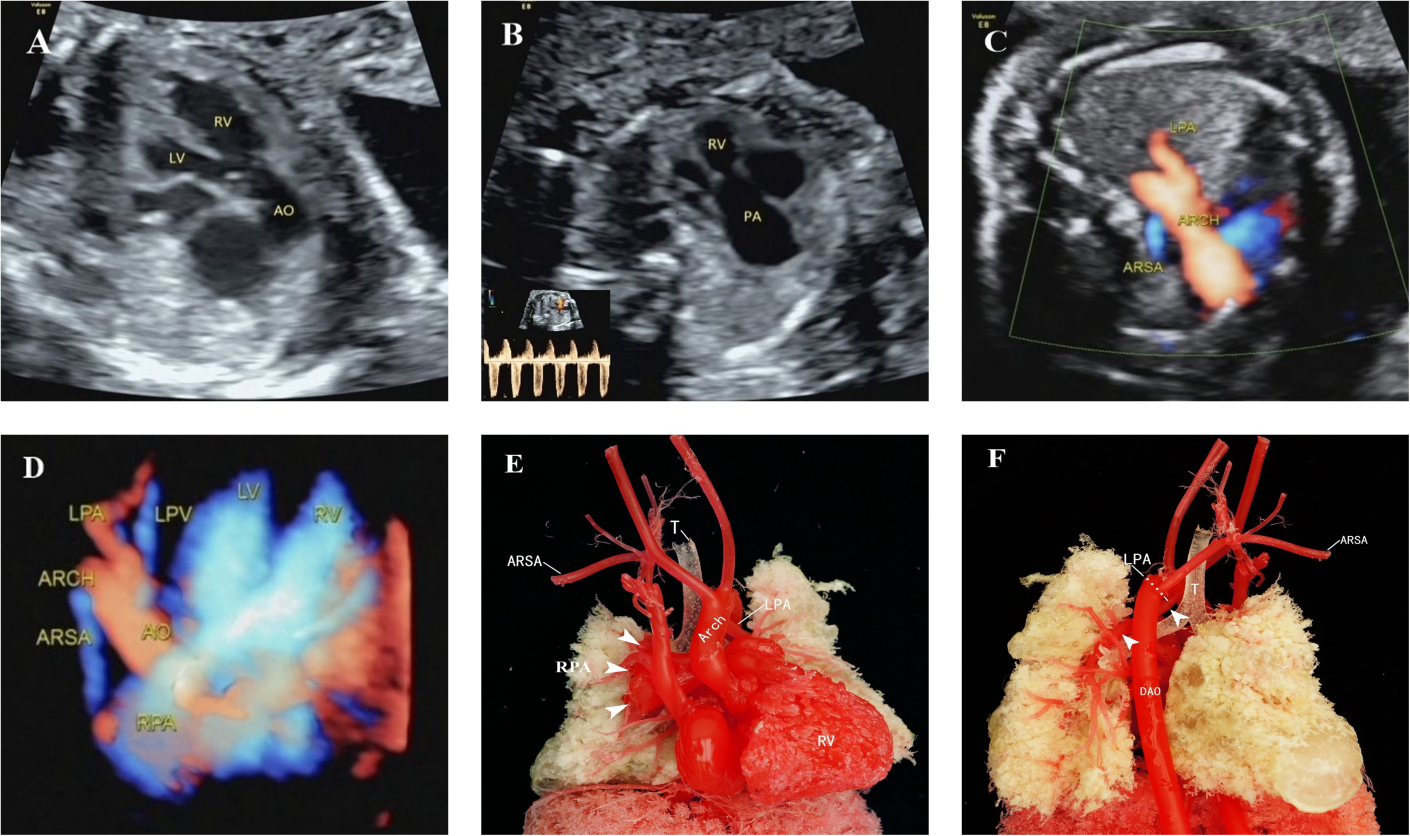
Unilateral absence of pulmonary artery with absent pulmonary valve in tetralogy of Fallot. (**A**) Outflow tract section showed the ventricular septal defect and aortic riding span. (**B**) Indistinct pulmonary valves, small development of the pulmonary annulus, significantly widened pulmonary artery and high-speed blood flow signal of two-way round trip in the spectrum Doppler. (**C,D**) Right subclavian artery was vagus, and the left pulmonary artery originated from the transverse arch of the aorta. (**E,F**) Anatomical casting showed that the absence of the pulmonary valve created a “whale’s tail” sign in the right pulmonary artery and that the left pulmonary artery originated from the transverse arch of the aorta. LV, left ventricle; RV, right ventricle; ARSA, aberrant right subclavian artery; LPV, left pulmonary vein; ARCH, aortic arch; DAO, descending aorta.

In this study, pregnancy termination was adopted for all five UAPA cases and pathological anatomy and casting confirmed the absence of the left pulmonary artery. The left pulmonary artery was attached to the innominate artery or the aortic arch. Interestingly, one case was complicated by the absence of a pulmonary valve and TOF, in which the anatomical casting showed that the absence of the pulmonary valve created a “whale’s tail” sign in the right pulmonary artery and that the left pulmonary artery originated from the transverse arch of the aorta (Figure 4).

### Missed and misdiagnosed conditions of the anomalous origin of the pulmonary artery

Of the four missed AOPA cases (4/26, 15.4%), one AORPA case (Case 5 in Table 3) and three PAS cases were missed. In the PAS population, two cases (Cases 11 and 12 in Table 2) were two sets of twins, with abnormality found in one of the twins for each set, and the remaining case (Case 13 in Table 2) was a singlet. They were all missed during the second-trimester screening and PAS was found by postnatal echocardiography. The AORPA case received a pathological autopsy and was complicated with multiple, severe intracardiac malformations.

Three UAPA cases were misdiagnosed (3/26, 11.5%), in which the absence of the left pulmonary artery was misdiagnosed as an anomalous origin of the left pulmonary artery branch from the aorta. They were all ultrasonically misdiagnosed as AOLPA during the second trimester and all opted for induction, in which one case (Case 3 in Table 4) was confirmed as UAPA by vascular casting and the other two cases (Cases 2 and 4 in Table 4) were confirmed as UAPA by pathological anatomy.

## Discussion

Prenatal ultrasonography can be adopted to observe the pulmonary artery on multiple sections as there is no gas interference in the lungs during the fetal period, which can depict the location, course, and inner diameter of the pulmonary artery throughout the process. The standard prenatal ultrasound examination may indicate the absence of a normal pulmonary artery bifurcation as a common ultrasonic feature of AOPA, in which one pulmonary artery extends directly from the main pulmonary artery and the pulmonary artery on the affected side is not detected. However, ultrasonic disparities remain regarding specific types of abnormalities. The pulmonary artery on the affected side of AOPA originates at the root of the ascending aorta or near the innominate artery and that of UAPA is connected to the aortic branch by ductus arteriosus, whereas the left pulmonary artery of PAS originates from the right pulmonary artery and travels posteriorly and leftward to the left hilus through the right side of the trachea. For AOPA incidences, color Doppler examination can reveal no connection between the abnormal origin of the pulmonary artery branch and the main pulmonary artery. Further spectral Doppler analysis may show that the blood vessels draining into the lung are characterized by the feature of the pulmonary artery spectrum. An important indicator of AOPA is the absence of one pulmonary artery on the pulmonary artery bifurcation section, in which the origin of the abnormal pulmonary artery should be detected in the right pulmonary artery, ascending aorta, or aortic arch branches and can be traced backward from the distal end of the pulmonary artery branch at the hilus on the affected side.

### Pulmonary artery sling

PAS is divided into the complete type and partial type (18). In the former, the main trunk of the left pulmonary artery originates from the right pulmonary artery. In the latter, although a rare case, part of the left pulmonary artery branches originates from the right pulmonary artery while the left pulmonary artery trunk and other branches remain normal in origin and course. A previous study reported a case of partial PAS in children (19), and the current study found 13 complete PAS cases. PAS is often complicated with segmental or extensive tracheal stenosis and tracheomalacia (20, 21), resulting in symptoms such as stridor, breath shortness, and recurrent lung infections. The clinical course and prognosis depend primarily on the degree and extent of airway narrowing. In this study, five of the 13 PAS cases reported tracheal narrowing, with one showing abnormal lung development due to severe bronchial stenosis. Therefore, when PAS is suspected during prenatal ultrasonography, the coronal section of the trachea should be scanned for abnormalities in morphology, course, and stenotic degree of the trachea. Available literature documents that about 40%–50% of PAS cases are complicated with other cardiovascular malformations (1) and that PAS can be associated with right ventricular myocardial insufficiency and total anomalous pulmonary venous connection (22, 23). In the current study, 61.5% of PAS cases were accompanied by other intracardiac malformations, such as PLSVC, TOF, and VSD.

With the recent development of high-resolution ultrasonic instruments and the accumulation of experience on the part of sonographers, the structure of fetal hearts can be better depicted during early pregnancy. In addition, nuchal translucency (NT) and ductus venosus flow spectrum can greatly improve the specificity and accuracy of screening for congenital cardiac malformations (24). In the current study, three of the thirteen PAS cases underwent fetal cardiac screening at 11–13^+6^ weeks, in which one case reported fetal NT thickening, reversed A wave in ductus venosus, and direct intracardiac signs of PAS; one case showed indirect signs, such as a right shift of the heart and increased left lung volume, and was diagnosed as PAS during middle and late pregnancy; and one case reported no obvious fetal heart structural abnormalities but was confirmed during the second trimester. Due to the small structure of the fetal heart during early pregnancy, as well as factors such as fetal position, the resolution of the instrument, and the expertise of the operator, an accurate diagnosis of PAS remains a challenge during early pregnancy, so a dynamic follow-up should be prescribed during the middle and late pregnancy.

Although PAS has typical antenatal echocardiographic features, it is prone to missed diagnosis. In this study, three PAS cases were missed, of which two were set of twins and one was a singleton. The potential explanations for the missed diagnosis may be as follows: (1) the relatively challenging and prolonged ultrasound examination for twins, which may bring about insufficiencies on the part of the technician and changes in the fetal position, thus resulting in the missed diagnosis; (2) the under-recognition of the importance of the three-vessel series section and insufficient knowledge of PAS; and (3) the adjacence of the fetal ductus arteriosus to the left pulmonary artery, which increases the chances of mistaking the ductus arteriosus for the left pulmonary artery.

### Anomalous origin of the unilateral pulmonary artery from the aorta

Available literature indicates a 5–6-fold higher incidence of AORPA than that of AOLPA (25). Consistently, the current study found, of the eight AOUPA cases in the enrolled study cohort, seven cases of AORPA and one case of AOLPA. Studies have demonstrated that after birth, the abnormal pulmonary artery receives a high-pressure blood flow from the aorta, while the unaffected pulmonary artery receives all the blood from the right ventricle, leading to pulmonary hypertension, obstructive pulmonary disease, and even heart failure (13). So, an early diagnosis and timely surgery are required to prevent irreversible vascular lung disease (26). If the initial part of the pulmonary artery originating from the ascending aorta is narrowed, gradual stenosis or even atresia may develop in that part of the unilateral pulmonary artery (1). In this study, two cases of the right pulmonary artery originating from the ascending aorta reported a narrowed start, in which the affected part deteriorated into the right pulmonary artery atresia after birth and the blood flow to the right lung was supplied by collateral blood vessels originating from the descending aorta. However, the transcollateral vessels were not revealed by CTA examination in Case 8. A possible explanation may lie in that the infant was young and the collateral blood vessels supplying the right lung were thin and could not be satisfactorily detected. Therefore, when the pulmonary artery is abnormally originating from the ascending aorta, attention should be paid to whether the beginning of the pulmonary artery is narrowed. Although AOUPA can be diagnosed early and repaired surgically, restenosis still may occur postoperatively (27).

In addition, AOUPA can be prone to other intracardiac structural abnormalities (28), such as TOF, CoA, interruption of the aortic arch, and VSD, which dictates huge prognostic disparities. Some studies suggest that AOLPA is more commonly associated with either TOF or the right aortic arch (25) and that AOLPA is more common than AORPA in TOF (28). In our study, the AOLPA case was complicated with TOF and the right aortic arch with mirror branches. Also, one of the AORPA cases was complicated by aortopulmonary window and disconnection of the aortic arch, which was diagnosed as Berry syndrome. The latter is an extremely rare complication of cardiovascular malformations, with an incidence of about 0.046% (29), mainly featuring distal aortopulmonary septal defect, dysplastic aortic arch (coarctation or disconnection of the aortic arch), a right pulmonary artery originating from the ascending aorta, and complete ventricular septum (30). Therefore, when AOUPA is detected prenatally, attention should be paid to the potential coexistence of other intracardiac malformations. The complications of other severe and frequent intracardiac malformations may mask the presence of AOUPA, resulting in missed diagnosis and misdiagnosis, and one case of AORPA was missed in this study. It is worth mentioning that we also need to distinguish AOUPA from persistent truncus arteriosus (PTA). AOUPA has separate pulmonary valves and a pulmonary artery trunk. In the PTA, the arteriosus also emits branches of the pulmonary artery, but the truncus arteriosus originating from the ventricle has only one set of semilunar valves.

### Unilateral absence of the pulmonary artery

UAPA is characterized by a disconnection between the main pulmonary artery and the pulmonary vessels in the lung parenchymal (the proximal unilateral pulmonary artery) but a connection between the distal part of the pulmonary artery and the intrapulmonary vessels. The distal pulmonary artery in the fetus is connected to the aortic arch branch or the transverse aortic arch via DA (12). In the UAPA group, five cases were reported. Due to the study protocol and the inclusion criteria of UAPA, we did not enroll cases of pulmonary atresia with ventricular septal defect (PA/VSD), unilateral pulmonary dysplasia, or absence because the vessels supplying the affected lung in the PA/VSD are collateral vessels from the descending aorta (31) and pulmonary agenesis is a complete absence of the pulmonary parenchyma, airways, and vasculature unilaterally or bilaterally (32). Therefore, they are not UAPA in the strict sense of the word. With the closure of DA on the affected side after birth, the distal part of the pulmonary artery in the lung also gradually atrophies or even deteriorates into atresia, forming a typical UAPA pathological change after birth (33). Unfortunately, all UAPA cases in this group chose to terminate the pregnancy, making unrealistic and impractical an analysis of postnatal UAPA fetuses. Although the absence of the right pulmonary artery is more common than the absence of the left pulmonary artery, the latter may occur more frequently if other cardiovascular malformations are combined (1). The missing pulmonary artery is usually located on the opposite side of the aortic arch (1). In this study, all five UAPA cases reported an absence of the left pulmonary artery, with blood supply via DA from the innominate artery or the transverse arch of the aorta, three of the left aortic arch, and two of the mirror branch of the right aortic arch.

UAPA is often associated with other congenital heart defects, of which TOF is the most common (15). The incidence of UAPA with TOF is about 1%–3% and that of the absent pulmonary valve (APV) with TOF is 5% (34). A complication of UAPA with TOF and APV is extremely rare. A previous study documents that the absence of the left pulmonary artery in 2% of patients with TOF is more frequent in 14.3% of patients with APVS (35). The study also reports one case of the absent right pulmonary artery with APVS (35). In our study, three of the five cases with the absent left pulmonary artery were complicated with TOF and one was coupled with TOF and APVS. Three UAPA cases were misdiagnosed as AOLPA, which may be attributed to the following considerations: (1) the location of the abnormal origin of the pulmonary arteries is both located in the distal end of the ascending aorta (immediately adjacent to the beginning of the innominate artery), which makes it difficult to accurately differentiate the distal AOUPA and UAPA before delivery; and (2) concomitant multiple cardiovascular malformations may confound the detection of the ductus arteriosus, which implies the necessity to detect the presence of DA.

## Limitation

This is a single-center study with a small sample size, and long-term outcomes are unavailable for analysis. Although this cohort reports a high induction rate and a frequent complication with multiple malformations, without genetic testing results, the causes of the anomalous origin of the fetal pulmonary artery cannot be determined. Future multicenter studies and further genetic verification are urgently awaited.

## Conclusions

Prenatal ultrasonography has a crucial value in diagnosing the anomalous origin of the fetal pulmonary artery. The absence of one pulmonary artery on the section of pulmonary artery bifurcation is an essential clue for diagnosing abnormal origins of the pulmonary artery. The location of the abnormal origin of the pulmonary artery on the affected side should be traced and attention should be paid to the potential complications with other intracardiac malformations. Pathological anatomy, postnatal CTA, and CT reconstruction may contribute to a better understanding of the anomalous origin of the pulmonary artery.

## Data Availability

The original contributions presented in the study are included in the article, further inquiries can be directed to the corresponding authors.
